# Elevated serum Neurofilament Light chain (NfL) as a potential biomarker of neurological involvement in Myotonic Dystrophy type 1 (DM1)

**DOI:** 10.1007/s00415-022-11165-0

**Published:** 2022-05-16

**Authors:** Tommaso F. Nicoletti, Salvatore Rossi, Maria Gabriella Vita, Alessia Perna, Gisella Guerrera, Federica Lino, Chiara Iacovelli, Daniele Di Natale, Anna Modoni, Luca Battistini, Gabriella Silvestri

**Affiliations:** 1 Dipartimento di Neuroscienze, Università Cattolica del Sacro Cuore, Largo F. Vito 1, 00168 Rome, Italy; 2 UOC Neurologia - Dipartimento Scienze dell’Invecchiamento, Neurologiche, Ortopediche e della Testa-Collo, Fondazione Policlinico Universitario A. Gemelli IRCCS, Largo Agostino Gemelli, 8, 00168 Rome, Italy; 3 Unità di Neuroimmunologia, Fondazione Santa Lucia IRCCS, Rome, Italy; 4 UOC Riabilitazione e Medicina Fisica–Dipartimento Scienze dell’Invecchiamento, Neurologiche, Ortopediche e della Testa-Collo, Fondazione Policlinico Universitario A. Gemelli IRCCS, Rome, Italy

**Keywords:** Myotonic dystrophy, Biomarkers, Central nervous system, Cognition, Neurofilament protein

## Abstract

**Background:**

Cognitive and behavioural symptoms due to involvement of the central nervous system (CNS) are among the main clinical manifestations of Myotonic Dystrophy type 1 (DM1). Such symptoms affect patients’ quality of life and disease awareness, impacting on disease prognosis by reducing compliance to medical treatments. Therefore, CNS is a key therapeutic target in DM1. Deeper knowledge of DM1 pathogenesis is prompting development of potential disease-modifying therapies: as DM1 is a rare, multisystem and slowly progressive disease, there is need of sensitive, tissue-specific prognostic and monitoring biomarkers in view of forthcoming clinical trials. Circulating Neurofilament light chain (NfL) levels have been recognized as a sensitive prognostic and monitoring biomarker of neuroaxonal damage in various CNS disorders.

**Methods:**

We performed a cross-sectional study in a cohort of 40 adult DM1 patients, testing if serum NfL might be a potential biomarker of CNS involvement also in DM1. Moreover, we collected cognitive data, brain MRI, and other DM1-related diagnostic findings for correlation studies.

**Results:**

Mean serum NfL levels resulted significantly higher in DM1 (25.32 ± 28.12 pg/ml) vs 22 age-matched healthy controls (6.235 ± 0.4809 pg/ml). Their levels positively correlated with age, and with one cognitive test (Rey’s Auditory Verbal learning task). No correlations were found either with other cognitive data, or diagnostic parameters in the DM1 cohort.

**Conclusions:**

Our findings support serum NfL as a potential biomarker of CNS damage in DM1, which deserves further evaluation on larger cross-sectional and longitudinal studies to test its ability in assessing brain disease severity and/or progression.

**Supplementary Information:**

The online version contains supplementary material available at 10.1007/s00415-022-11165-0.

## Introduction

Myotonic Dystrophy type 1 (DM1), also known as Steinert disease (OMIM #160,900), is the most common muscular dystrophy in adults, showing a prevalence of ~ 1:8000 among Caucasians. It is caused by an unstable expansion of a CTG trinucleotide repeat located at the 3′ UTR of *DMPK* gene (OMIM #605,377) in a pathological range from 50 to several thousand of repeats [1, 2]. Mitotic and intergenerational instability of pathological CTG expansions underlie the interindividual clinical variability and the anticipation phenomenon during parent to child transmission, respectively [3].

In DM1, the range of n(CTG) determined in peripheral leukocytes inversely correlates with age at onset of symptoms, which in turn is associated with distinct severity of DM1 clinical presentations: these include the most severe congenital (symptoms present at birth), then infantile (1–10 years), juvenile (11–20 years), adult (21–40 years), and finally oligosymptomatic late-onset form (> 40 years) [4]. The major pathogenic effect related to the pathological CTG expansion is the ubiquitous transcription of CUG expanded pre-mRNAs, which accumulate within the nucleus, eventually producing transdominant splicing defects on other genes [5]. This process is at the basis of the typical multisystem involvement in DM1, also affecting the central nervous system (CNS) [1, 2].

Cognitive and behavioural symptoms related to CNS involvement are among the main clinical manifestations in DM1 [6]: congenital and infantile forms manifest with intellectual disability, suggestive of a neurodevelopmental defect, whereas focal involvement of fronto-temporal cognitive functions occurs in juvenile/adult and late-onset forms. Such distinct pattern of cognitive defect grossly correlates with the n(CTG) expansion in leukocytes, as both congenital and infantile DM1 forms harbour larger CTG expansions than juvenile/adult and late-onset patients [4].

In DM1 patients, brain MRI document widespread grey and deep white matter alterations, especially involving the frontal and temporal lobes [7], and accordingly, FDG-PET and SPET studies demonstrate hypometabolism in the same areas [8, 9]. Neuropathology of DM1 brains show variable grey and white matter atrophy, neurofibrillary degeneration associated with aberrant *MAPT RNA* splicing, myelin loss, and gliosis [10].

Current development of gene-based therapies for DM1 able to reduce intracellular toxic RNAs and restore normal gene expression might hopefully move toward their short-term translation into human clinical trials [11]. Therefore, search for sensitive, prognostic biomarkers of CNS involvement is crucial to assess their effectiveness also on brain tissue damage. So far, many studies evaluated the role of neuroimaging, particularly high-resolution 3 T brain MRI, as a prognostic biomarker of CNS damage, yet their results have been controversial [7]. Recently, CSF and circulating Neurofilament Light chain (NfL) has been assessed as a sensitive and reliable prognostic and monitoring biomarker in various CNS disorders [12]. Its measurement in blood makes possible easy and repeated assessments also for monitoring disease course, being an ideal outcome tool, particularly in DM1 patients. Therefore, we conducted a study aiming to investigate the role of serum NfL as a potential biomarker of CNS involvement also in DM1. The primary aim was to evaluate if serum NfL levels are significantly elevated in DM1 patients compared to healthy controls. Secondary aims were to assess any associations and/ or correlations between serum NfL levels and cognitive defects, white matter alterations at brain MRI, other clinic-diagnostic features (disease form and duration, skeletal muscle and respiratory involvement, and nCTG in leukocytes), and demographic parameters in DM1 patients.

## Materials and methods

This study was designed and carried out in compliance with standards of the Helsinki Declaration, and of the Good Clinical Practice, and approved by the Local Ethical committee (ID 2665). All participants gave a written informed consent to the study.

We enrolled a cohort of consecutive 40 patients ≥ 18 years of age with proven molecular diagnosis of DM1, among those diagnosed and in follow-up in our Neurological Tertiary Centre for Neuromuscular Diseases. Besides DM1 molecular diagnosis, inclusion criteria were: normal kidney and thyroid functions, capacity to perform neuropsychological tests, no history of traumatic brain injury, stroke, epilepsy, and other not DM1-related brain lesions.

### Measurement of NF-light levels

Eight ml samples of peripheral blood collected from each DM1 patient were centrifuged within 3 h from sampling for 15 min at 3000 revolutions per minute (rpm) for serum separation, that was immediately stored at  – 80 °C. Frozen serum samples were then carried in dry ice to the Neuroimmunology Lab, Santa Lucia Foundation IRCCS, Rome (IT) for quantitative determination of NfL using an ultrasensitive immunoassay on the Single-Molecule Assay (SiMoA) platform [13]. Briefly, the assay was performed using the commercially available NF-light Advantage (SR-X) kit (Quanterix, item 103,400), run on the fully automated ultrasensitive SiMoA SR-X Analyzer (Quanterix Corporation, Massachusetts), following a two-step digital protocol. Calibrators (neat) and serum samples were measured in duplicate in accordance with the manufacturer’s instructions with appropriate standards and internal controls. Dynamic range of detection was from 0 pg/mL to 2000 pg/mL for measurements of serum samples. NfL levels from the DM1 group were compared with those obtained in a control group including 22 age- and sex-matched healthy subjects.

### Diagnostic data for statistical analysis

In 32 out of 40 DM1 patients (80.0% of total cases), we collected neuropsychological data performed within 6 months from blood sample collection for NfL measurement. The cognitive test battery included the Mini-Mental State Examination (MMSE) [14], the Rey's Auditory Verbal Learning Task (RAVLT) including subtests of immediate and delayed recall and forced-choice recognition [15], the Rey–Osterrieth Fig. copy and delayed recall [16], an abstract reasoning test (Raven's Progressive Matrices) [15], the Stroop test–short version [15], a demanding visual attention task (Multiple Features Target Cancellation) [17, 18], phonological [15] and semantic verbal fluency [19], an objects naming task [15], and digit and spatial span forward and backward [20]. This neuropsychological protocol allowed to assess all cognitive domains, especially those regarding frontal functions and linguistic abilities subserved by temporal areas. Of note, most selected tests did not require manual skills to rule out a bias due to the presence of patients’ hand muscle weakness.

Presence and severity of brain white matter alterations were assessed by the Fazekas score [21] in 19 out of 40 DM1 patients, who had performed a diagnostic brain MRI within 12 months before or 3 months after the blood sampling for NfL analysis (Table 1).

**Table 1:** Demographic and clinical characteristics of DM1 patients

	Count (%)	Median	Min	Max	Mean	SD	*n*
Male	25 (62.5)						40
CTG repeat lenght		575	75	1400	625.13	376.65	38
Disease form:							
Congenital	1 (2.5)						
Infantile	8 (20)						
Juvenile/adult	28 (70)						
Late-onset	3 (7.5)						
Disease severity (MIRS score)		3.5	1	5	3.35	0.83	40
Disease duration (years)		27.7	2.58	51.92	26.5	12.2	40
Age at onset		19	1	60	21.2	13.4	40
NfL levels (pg/ml)		15.58	3.47	122.23	25.32	28.12	40
Age at examination (years)		47.8	23.83	69.17	47.7	10.8	40
Education (years)		11.5	8	18	11.32	3.18	40
NIV support indication	22 (62.9)						35
NIV compliance	13 (59.1)						22
FVC (%)		70	40	118	70.72	22.49	25
Fazekas score:							
0	3 (15.8)						
1	13 (68.4)						
2	1 (5.3)						
3	2 (10.5)						
Polyneuropathy	1 (2.5%)						40

Abbreviations: *MIRS* muscular impairment rating scale, *NfL* neurofilament light chain, *NIV* non-invasive ventilation, *FVC* forced vital capacity, *SD* standard deviation

Following available data in DM1 patients were also collected for the statistical analysis (Table 1): age, sex, DM1 clinical form (i.e., congenital, infantile, juvenile/adult/late-onset), years of disease duration, muscle disease severity assessed by the Muscular Impairment Rating Scale (MIRS) [22], n(CTG) in peripheral leukocytes, years of education, indication and compliance to non-invasive ventilation (NIV) for respiratory problems (either sleep disordered breathing (SDB) or restrictive pulmonary syndrome), % pulmonary forced vital capacity (FVC), and presence of polyneuropathy (this last item was included as its presence might influence NfL levels determination).

### Statistical analysis

The sample was characterized in its clinical and demographic features using descriptive statistics techniques. Quantitative variables were described using mean and standard deviation (SD). Qualitative variables were summarized with absolute and percentage frequency tables. Normality of continuous variables was checked using Shapiro–Wilk probability test. N-value was specified for each variable. Patients with some missing values have been included in the study and maintained as missing. Mann–Whitney *U* Test was used to compare NfL levels between controls and patients, between males and females (only in the cohort of DM1 patients) and between patients with normal cognitive profile and patients with at least one pathological cognitive task. The Spearman correlation test was used to evaluate correlations between NfL levels and the other collected variables. Significance level was set at *p* < 0.05. Statistical analysis was performed by SPSS (Statistical Package for Social Science, IBM SPSS Statistics, Version 24.0. Armonk, NY: IBM Corp).

## Results

Demographic and clinical characteristics of the study population are summarized in Table 1. The cohort of 40 DM1 patients enrolled into the study had a mean age of 47.7 ± 10.8 years, with a moderate male prevalence (25/40, 62.5%). The study cohort included 1 congenital, 8 childhood, 28 juvenile/adult, and 3 late-onset DM1 individuals. Mean age at onset was 21.2 ± 13.4 years, disease duration was 26.5 ± 12.2 years, and n(CTG) obtained at the time of diagnosis of DM1 was 625.13 ± 376.65. Mean MIRS score was 3.35 ± 0.83, indicating an overall moderate, mainly distal, muscle involvement. Only 1 DM1 patient had an associated mixed sensory–motor polyneuropathy. The control group included 22 age- and sex-matched individuals (mean age 45.6 ± 9.8 ys, 12 males).

**Fig. 1:**
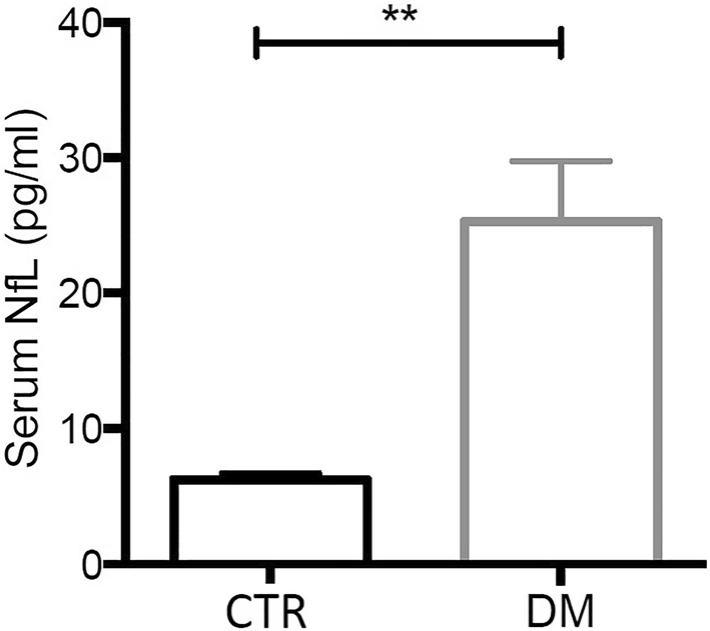
Histogram showing mean levels of Neurofilament Light chain (NfL) [pg/ml] in DM1 patients (*n* = 40) vs controls (CTR, *n* = 22). Asterisks (**) indicate significant difference (*p* = 0.0024) by Mann–Whitney *U* test

Mean NfL levels resulted significantly elevated in DM1 patients (25.32 ± 28.12 pg/ml) compared to controls (6.235 ± 0.4809 pg/ml, p = 0.0024) (Fig. 1). Of note, 37 out of 40 DM1 patients (92.5%) showed serum NfL levels above the normal range (5–7 pg/ml) according to the literature [23].

Neuropsychological tests documented the presence of cognitive impairment, consisting of a mild-to-moderate fronto-temporal dysfunction, in 25 out of 32 DM1 patients (78.1%). The most frequently altered tests were: the copy and delayed recall of Rey’s complex Fig. (40.6% and 53.1%, respectively), spatial and verbal working memory tests (31.3% and 28.1%, respectively), the Stroop test execution time (25%), a phonological verbal fluency test (18.8%), and a picture-naming task concerning objects (18.8%). Descriptive statistics of neuropsychological tests scores obtained in DM1 patients are illustrated in Table 2. The majority of DM1 patients with available neuroimaging had a Fazekas score = 1 (68.4%, 13/19, Table 1), indicating the presence of mild multiple punctate lesions affecting white matter at routine brain MRI.

**Table 2:** Neuropsychological profile of the DM1 patients

	Median (adjusted)	Min (adjusted)	Max (adjusted)	Mean (adjusted)	SD (adjusted)	Impaired in %	*n*
MMSE	28.00 (27.03)	17.00 (16.60)	30.00 (30.00)	27.08 (26.58)	3.24 (3.11)	15.6 (5)	32
RAVLT immediate recall	46.50 (39.20)	16.00 (24.99)	69.00 (59.78)	46.22 (42.35)	10.10 (9.06)	9.4 (3)	32
RAVLT delayed recall	10.00 (9.49)	5.00 (3.80)	15.00 (13.45)	10.53 (9.30)	2.46 (2.24)	3.1 (1)	32
RAVLT forced-choice recognition	(0.97)	(0)	(1.00)	(0.92)	(0.18)	12.5 (4)	32
Rey’s complex figure recall	12.25 (9.19)	1.00 (0)	32.50 (27.65)	12.80 (9.96)	7.51 (7.52)	53.1 (17)	32
Digit span forward	5.00 (4.90)	3.00 (2.44)	6.00 (5.91)	5.09 (4.83)	0.86 (0.83)	18.8 (6)	32
Digit span backward	3.00 (3.19)	0 (0)	5.00 (4.83)	3.38 (3.13)	1.01 (0.99)	31.3 (10)	32
Spatial span forward	4.00 (4.06)	0 (0.82)	7.00 (6.54)	4.41 (4.22)	1.27 (1.17)	18.8 (6)	32
Spatial span backward	4.00 (3.57)	0 (0.29)	6.00 (5.81)	3.94 (3.62)	1.24 (1.24)	31.3 (10)	32
Raven’s colored progressive matrices	27.00 (26.57)	10.00 (13.30)	35.00 (35.30)	26.31 (25.10)	5.92 (5.84)	12.5 (4)	32
Rey’s complex figure copy	30.00 (29.13)	3.50 (6.36)	36.00 (35.46)	27.92 (26.97)	8.38 (7.94)	40.6 (13)	32
MFTC accuracy	(0.92)	(0)	(1.00)	(0.89)	(0.19)	15.6 (5)	32
MFTC time	60.00 (57.39)	30.00 (17.14)	180.00 (194.11)	62.56 (63.54)	31.89 (35.75)	9.4 (3)	32
Phonological verbal fluency	31.50 (29.75)	3.00 (4.41)	52.00 (50.10)	31.25 (29.00)	12.20 (11.71)	18.8 (6)	32
Categorical verbal fluency	19.50 (16.77)	8.00 (2.38)	48.00 (49.90)	20.34 (17.51)	9.12 (10.76)	21.9 (7)	32
Naming of pictures of objects	30.00	14.00	30.00	28.03	3.57	18.8 (6)	32
Stroop test time	28.25 (29.50)	8.50 (13.76)	66.00 (65.20)	29.20 (31.15)	12.46 (12.69)	25.0 (8)	32
Stroop test errors	0.50 (1.04)	0 (0)	0.95 (8.70)	1.53 (1.55)	2.24 (2.06)	12.5 (4)	32

Abbreviations: *MMSE* mini-mental state examination, *RAVLT* rey auditory verbal learning test, *MFTC* multiple features targets cancellation, *SD* standard deviation

**Fig. 2:**
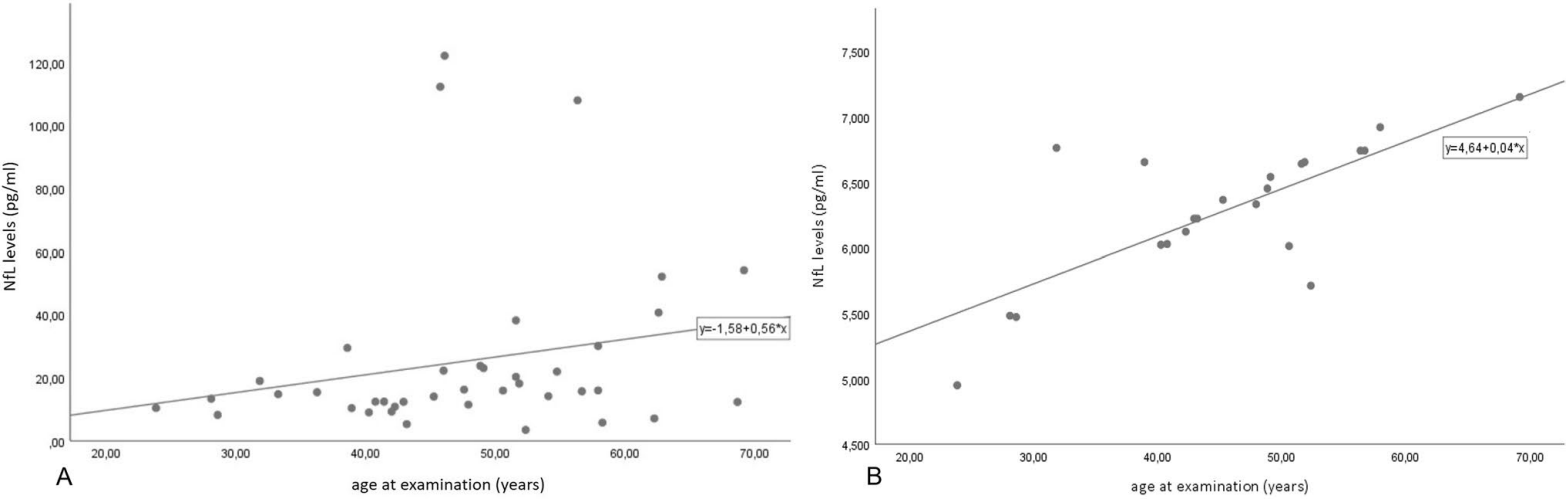
Panel displaying in **A** scatter plot with linear regression showing correlation between Neurofilament Light chain (NfL) levels [pg/ml] and patients’ age at examination (*n* = 40), and **B** corresponding scatter plot of controls (*n* = 22)

In the DM1 cohort, statistical analysis documented a positive correlation between NfL levels and age at examination (*p* = 0.049, ρ = 0.314 Fig. 2A), while no significant differences were found regarding NfL levels between male and female DM1 patients. As expected, a significant positive correlation between NfL levels and age was found also in controls (*p* = 0.003, ρ = 0.609, Fig. 2B).

Considering cognitive performances, NfL levels inversely correlated only with the scores at RAVLT’s immediate recall and recognition tasks (Table 3-Supplementary material). Also, comparison between DM1 patients with normal cognitive profile (8 out of 33) vs DM1 patients with at least one pathological cognitive task (25 out of 32) showed no significant differences in serum NfL levels between the two groups (data not shown). Serum NfL levels also directly correlated with Fazekas score (*p* = 0.012, ρ = 0.562, Table 3-Supplementary material), whereas they did not correlate with MIRS, n(CTG), DM1 clinical form, age at onset, disease duration, NIV treatment, or %forced vital capacity (FVC).

## Discussion

Cognitive and neuropsychiatric manifestations are one of the main disabling features of DM1 patients [24], which can remarkably impact on their personal and family life quality, and on disease prognosis [25]. Increasing knowledge about the pathogenesis of DM1 [26] is now raising concrete therapeutic perspectives able to correct aberrant splicing [5, 11]. However, several issues complicate design of clinical trials to test safety and efficacy of these promising drugs in DM1. First, the variability in the clinical presentation challenges definition of the natural history of the disease. Second, the cognitive and behavioural symptoms in DM1 patients affect the reliability of already validated subjective and objective outcome measures [27] and can lead to patients’ selection bias in study cohorts.

Given the relevance of clinical CNS manifestations in DM1, many neuroimaging studies aimed to define underlying brain structural and functional alterations and to assess neuroimaging as a potential prognostic biomarker of CNS involvement [28].

The role of high-resolution 3 T brain MRI as a prognostic biomarker of CNS damage is still debated, because of conflicting results possibly related to the limited sample of the study cohorts and to differences in the study protocols [28]. Moreover, a few performed longitudinal studies [29, 30] suggested that high-resolution MRI could detect significant progression of brain damage in DM1 only after many years of follow-up. Brain MRI might not be an ideal CNS biomarker in DM1 also because patients’ compliance might be affected by severe cognitive/behavioural symptoms, respiratory problems impairing prolonged supine position, or implanted therapeutic devices (i.e., lenses, pacemaker, or implantable cardioverter defibrillator), causing enrolment bias or study drop-out.

The availability of a circulating biomarker of CNS damage in DM1 could overcome such critical aspects, so we decided to perform a study to assess the potential role of NfL as a biomarker of CNS involvement in DM1. Neurofilaments (Nf) are neuronal-specific heteropolymers, composed of triplet of light (NfL), medium (NfM), and heavy (NfH) chains. In mature axons, NF represent the most copious proteins: in healthy subjects, NF undergo constant degradation and renewal, with consequent release into the CNS interstitial space and then in the bloodstream, so that cerebrospinal fluid and serum concentrations strongly correlate [31]. Therefore, determination of plasma or serum NfL is now considered the candidate marker of outcome in several neurological disorders, correlating with the intensity of neuroaxonal damage [12] and useful to assess response to treatment [32]. For serum NfL determination, we used the SiMoA technique, which allows to sufficiently measure the single-digit picogram/mL in the blood, increasing its sensitivity compared to other immunoassays [13].

Our results show that serum NfL levels are significantly higher in DM1 patients, with mean values four times more elevated than healthy controls (Fig. 1). To note is that our cohort presented a relatively young mean age (47.7 ± 10.8 years), ruling out the contribute of physiological aging or vascular damage to NfL release, frequently observed in individuals over 60 years of age [33]. These findings agree with lately published data from other research groups, which documented significantly elevated serum NfL levels either in smaller [34, 35] and comparable DM1 cohorts [36], and overall support serum NfL levels as a marker of neuroaxonal damage in DM1. Of note, measurement of NfL levels can assess neuroaxonal damage related to various etiologies in DM1, such as aberrant splicing of *MAPT* or other genes regulating synaptic integrity, neurotransmission and neuroinflammation [37], and sleep disordered breathing [38].

Correlation analysis regarding circulating NfL levels in this study was mainly focused on the cognitive involvement, being one of the main CNS manifestations in DM1 [24]. Besides a positive correlation between NfL and patients’ age at examination, already described in the literature [14], in our study cohort, NfL levels inversely correlated with the scores at RAVLT’s immediate recall and recognition tasks, while no correlation was observed with performances at delayed recall of the RAVLT. These findings may depend on the fact that in DM1 patients, episodic memory impairment is mainly related to attentional and executive alterations during the learning and recognition phases. In fact, as widely documented [24, 39] and confirmed by our data, the cognitive profile in juvenile/adult-onset DM1 typically displays attentional and executive impairment, while episodic memory is usually involved either in patients at more advanced disease stages or in those manifesting with late-onset forms [39]. On the other hand, serum NfL did not show any correlations with other cognitive performances, differently from what found on cohorts of patients’ with neurodegenerative dementias [12]. Such difference may reasonably depend on the smaller sample size of this study cohort, due to the rarity of DM1 compared to degenerative dementias. Also, this lack of correlation may depend on the fact that our cohort did not equally cover the entire DM1 clinical spectrum, as most of the patients were juvenile/adult forms (Table 1). Nevertheless, we point out that 2 out of 3 patients with the highest serum NfL values were manifested with congenital/infantile form, that is in fact characterized by global intellectual disability, whereas 2 out of 3 patients with normal serum NfL showed normal cognitive performances. This observation supports that NfL levels might actually reflect the global severity of cognitive impairment in DM1.

We should also take into account that, in our DM1 cohort, we did not perform an assessment of behavior or personality traits, symptoms likely resulting from a functional axonal damage which might also significantly contribute to increased NfL release. Indeed, Nfl are also integral components of synapses and contribute to modulate neurotransmission and behavior in vivo [40]. Accordingly, increased circulating NfL levels have been reported in patients with primary psychiatric diseases, such as major depressive disorders and schizophrenia [41], whose pathogenesis appears to be related to an abnormal brain connectivity [42].

Thus, in DM1 patients, increased NfL release might be also consequent to the known disruption of specific neural networks regulating mood and/or behavior [43–51]. In this regard, in a very recent study [36], circulating Nfl levels in DM1 patients also correlated with white matter DTI changes, a measure of abnormal brain connectivity [36], and accordingly, we found a correlation between Nfl levels and the Fazekas score, an outcome measure of structural brain MRI white matter changes, confirming that white matter damage would be predominant in DM1 brains [28].

Finally, in our cohort study, serum NfL levels did not correlate with other DM1 diagnostic parameters, particularly those reflecting skeletal muscle involvement, such as MIRS score and respiratory function tests, and with the n(CTG) in leukocytes. Beside the limited sample size, this issue is likely due to the occurrence of somatic mosaicism in DM1 tissues, leading to differences either in size or tissue stability of the CTG repeat over time [3]; in fact, in DM1 patients’ estimation of the progenitor allele (ePAL) length by small pool PCR might be a better predictor of CNS involvement measured by NfL than CTG expansion sizing by long PCR [36].

In conclusion, the results of our study, together with other very recent literature reports, support serum NfL levels as a potential biomarker of CNS involvement in DM1. Further cross-sectional and longitudinal studies on larger DM1 cohorts including comparable subgroups of clinical forms and assessment of metacognitive and psychiatric manifestations are needed to confirm if serum NfL might represent a sensitive prognostic and monitoring outcome tool as regards brain involvement in DM1.

## Supplementary Information

Below is the link to the electronic supplementary material.

Supplementary file1 (DOCX 18 KB)
