# Supplementary material for: Elevated serum Neurofilament Light chain (NfL) as a potential biomarker of neurological involvement in Myotonic Dystrophy type 1 (DM1)
Source: J Neurol. 2022 May 16;269(9):5085–92. doi: 10.1007/s00415-022-11165-0 (PMC9363395; doi:10.1007/s00415-022-11165-0)
Supplement: Supplementary file 1 — Supplementary file1 (DOCX 18 KB) [file 415_2022_11165_MOESM1_ESM.docx]

**Table 3.** Correlation between serum Neurofilament Light Chain (NfL) and the other collected variables (cognitive performance scores and clinical data). Spearman correlation coefficient and P value are reported (the latter one between brackets). Significant correlations are highlighted in bold. Abbreviations: MMSE: Mini-mental state examination; RAVLT: Rey’s Auditory Verbal Learning Test; MFTC: Multiple Features Target Cancellation; FVC: Forced Vital Capacity; MIRS: Muscular Impairment Rating Scale.

|  |  | **NfL** |
| --- | --- | --- |
| Cognitive Performance | MMSE | -0.154  (0.400) |
|  | RAVLT _immediate recall_ | **-0.413**  **(0.019)** |
|  | RAVLT _delayed recall_ | -0.131  (0.476) |
|  | RAVLT _forced-choice recognition_ | **-0.549**  **(0.001)** |
|  | Rey’s complex figure recall | -0.141  (0.441) |
|  | Digit span forward | -0.159  (0.385) |
|  | Digit span backward | -0.243  (0.180) |
|  | Spatial span forward | 0.010  (0.959) |
|  | Spatial span backward | 0.113  (0.538) |
|  | Raven’s Matrices | -0.043  (0.816) |
|  | Copy of Rey’s complex figure | -0.022  (0.906) |
|  | MFTC _accuracy_ | -0.260  (0.151) |
|  | MFTC _time_ | -0.028  (0.880) |
|  | Phonological verbal fluency | 0.019  (0.916) |
|  | Semantic verbal fluency | 0.072  (0.696) |
|  | Naming of pictures of objects | 0.010  (0.956) |
|  | Stroop _time_ | -0.254  (0.160) |
|  | Stroop _error_ | 0.023  (0.900) |
| Clinical Data | n(CTG) | -0.240  (0.147) |
|  | Age at disease onset | 0.196  (0.225) |
|  | FVC | -0.102  (0.629) |
|  | Fazekas score | **0.562**  **(0.012)** |
|  | MIRS score | 0.025  (0.876) |
